# American College of Radiology thyroid imaging report and data system combined with *K-RAS* mutation improves the management of cytologically indeterminate thyroid nodules

**DOI:** 10.1371/journal.pone.0219383

**Published:** 2019-07-11

**Authors:** Hongxun Wu, Bingjie Zhang, Gangming Cai, Jie Li, Xiaobo Gu

**Affiliations:** 1 Department of Ultrasound, Jiangyuan Hospital Affiliated to Jiangsu Institute of Nuclear Medicine (Key Laboratory of Nuclear Medicine, Ministry of Health/Jiangsu Key Laboratory of Molecular Nuclear Medicine), Wuxi, Jiangsu, China; 2 Clinical Molecular Biology Laboratory, Jiangyuan Hospital Affiliated to Jiangsu Institute of Nuclear Medicine (Key Laboratory of Nuclear Medicine, Ministry of Health/Jiangsu Key Laboratory of Molecular Nuclear Medicine), Wuxi, Jiangsu, China; Medical University of Vienna, AUSTRIA

## Abstract

We investigated whether use of American College of Radiology thyroid imaging report and data system (ACR TIRADS) in combination with *K-RAS* mutation status may facilitate risk stratification of patients with cytological Bethesda Category III and IV thyroid nodules. Ultrasonographic, cytological, and histopathological diagnoses were retrospectively correlated with *K-RAS* mutation status in a series of 43 cytologically indeterminate thyroid nodules (CITNs) that were referred for surgical excision. *K-RAS* mutations were detected in 8/43 (18.6%) fine-needle aspiration (FNA) samples as against 11/43 (25.6%) surgical specimens. ACR TIRADS level (TR) TR3 lesions had a malignancy risk of 40%; the *K*-*RAS* mutation rate in FNA samples and surgical specimens of category TR3 lesions was 40% and 60%, respectively. *K-RAS* mutation-positive malignancy was significantly more frequently detected in follicular neoplasm/suspicious for follicular neoplasm (FN/SFN) lesions than that in atypia or follicular lesion of undetermined significance (AUS/FLUS) (P<0.01). Combined use of ACR TIRADS (TR5 as the diagnostic threshold) and *K-RAS* mutation status helped identify 83.3% (10/12) malignant nodules (58.6% specificity, 45.5% positive predictive value, 89.5% negative predictive value, and 65.9% accuracy). CITNs with ACR TIRADS category TR3 showed an unexpectedly high risk of malignancy. *K-RAS* mutation-positive FN/SFN nodules have a 50% risk of malignancy and surgery should be recommended. Combined use of ACR TIRADS and *K-RAS* mutation may facilitate risk-stratification of patients with CITNs. The high negative predictive value (NPV) for malignancy seems sufficient to allow conservative management of patients with active surveillance.

## Introduction

Thyroid nodules are common in the general population with a malignant rate of 5–15% [1]. Ultrasonography (US) is currently recommended as the initial modality for evaluation and workup of thyroid nodules. However, the diagnostic accuracy is heavily dependent on the experience and ability of the operator to describe and interpret suspicious features. In order to minimize the interobserver variability, use of a series of thyroid imaging report and data system (TIRADS) was proposed for classification and malignant risk-stratification of thyroid nodules [2–4]. Fine-needle aspiration (FNA) cytology is regarded as the most efficient method for evaluation of thyroid lesions [5]. However, the cytology results may be indeterminate in up to 25% of thyroid nodules [5–6]. There is no consensus on the diagnosis and management of cytological indeterminate thyroid nodules (CITNs), i.e., Bethesda Category III lesions [atypia of undetermined significance/follicular lesion of undetermined significance (AUS/FLUS)] and Category IV [follicular neoplasm/suspicious for follicular neoplasm (FN/SFN)]. Moreover, the reported risk of malignancy in CITNs shows wide variability [5–7]. Owing to this ambiguity, appropriate management of CITNs is a clinical challenge. Active surveillance or diagnostic surgical resection is recommended for such cases based on the clinical and US features [7]. Investigation of molecular makers is also a useful adjunctive tool for diagnosis of CITNs [7]. *BRAF* and *RAS* point mutations are currently the most widely used markers for CITNs. *RAS* mutations are not specific for malignancy and have a lower positive predictive value than *BRAF*; however, *RAS* mutation status may provide useful supplemental information for management of CITNs. However, to the best of our knowledge, no study has evaluated the application of ACR TIRADS in combination with *RAS* mutation analysis for evaluation of CITNs. Therefore, the purpose of this study was to evaluate the performance of the ACR TIRADS in combination with *RAS* mutation status for identification of potential malignant thyroid nodules with indeterminate cytology.

## Material and methods

### Study population

The study protocol was reviewed and approved by the Institutional Review Board of Jiangsu Institute of Nuclear Medicine. The requirement for written informed consent of patients was waived off. Between January 2017 and June 2018, 2074 patients underwent preoperative US-guided fine-needle aspiration of thyroid nodules at the department of Ultrasound, Jiangyuan Hospital affiliated to Jiangsu Institute of Nuclear Medicine (Wuxi, China). Patients who qualified the following criteria were included in this study: (1) indeterminate thyroid cytology based on the Bethesda system; (2) histological diagnosis confirmed by surgical resection; (3) availability of *BRAF* and *K*-*RAS* mutation status; (4) patients received an ultrasound examination of the thyroid, which was classified by ACR TIRADS. Finally, a total of 43 patients (9 male, 34 female) with 43 CITNs were included in the analysis. Anonymized patient information was acquired and stored in a research database before accessed.

### US examination and ACR TIRADS

All US examinations were performed using a 5–12 MHz linear-array probe (iU22, Philips Healthcare, Bothell, WA, USA) by 1 of 10 radiologists who had 3 to 23 years of experience in thyroid imaging. A radiologist with 21 years of experience in thyroid imaging, blinded to cytopathological and histopathological diagnosis, retrospectively reviewed the US features of all CITNs.

The composition of the nodules was classified as solid or almost completely solid, mixed cystic and solid, cystic or almost completely cystic, and spongiform. Echogenicity was categorized as hyperechoic or isoechoic, hypoechoic and very hypoechoic. The shape of the nodule was classified as taller than wide (anteroposterior diameter greater than the transverse or longitudinal diameter) or wider than tall. The nodule margin was categorized as smooth, ill-defined, lobulated or irregular, or with extra-thyroid extension. Calcification was categorized as none or large comet-tail artifacts, macrocalcification, peripheral calcification, or punctuate echogenic foci. Subsequently, ACR TIRADS, a five-tier risk categorization system, was assigned to each nodule based on the aforementioned US features. TIRADS points were also recorded for each nodule.

### US-guided fine-needle aspiration and detection of molecular markers

Written informed consent was obtained from all patients undergoing ultrasound-guided fine needle aspiration (UG-FNA). Following the recommendations given by surgeons, four patients with category TR2 nodules underwent UG-FNA because of enlargement of solid portion. All thyroid FNA specimens were collected under ultrasound guidance using a 23-gauge needle attached to a 2.5-mL disposable plastic syringe by a radiologist using the freehand technique. Three passes were performed for each thyroid nodule. The slides were immediately placed in 95% ethanol for Papanicolau staining. On-site cytological assessment was not available. Two of the three cytopathologists specializing in thyroid pathology interpreted the cytology reports based on the Bethesda System for Reporting Thyroid Cytopathology. After cytological preparation, the residual biopsy specimen in the syringe was used for *BRAF*^V600E^ and *K*-*RAS* mutation analysis. *BRAF*^V600E^ mutation analysis in FNA samples is routinely performed as an ancillary test at our institute, and *K*-*RAS* mutation analysis is performed only in case of indeterminate cytological samples.

Molecular biology techniques were processed as standard technique for clinical pathology lab. The needle tip was washed with 100 μL Buffer ATL and residual FNA material was pipetted directly into a microcentrifuge tube for molecular analysis. DNA was isolated using the QIAamp DNA Micro kit (Qiagen), according to the manufacturer’s instructions. DNA extraction from formalin-fixed paraffin-embedded tissue specimens was performed as described elsewhere [8]. The methodology for analysis of BRAFV600E mutation is described elsewhere [9]. *K-RAS* mutation status was determined using real-time polymerase chain reaction (PCR) amplification followed by high resolution melting (HRM) analysis. The melting curve was analyzed by LightCycler 480 software (Roche Diagnostics). The position of the melting peaks was different for mutated-type and wild-type. Melting temperature (Tm) was identified by the peak position of the melting curve.

### Statistical analyses

Sample size calculations had been made before gathering data. Finally, the sample size was determined by the feasibility of recruitment, and it was justified by minimally detectable effect sizes instead of power analysis. Comparisons of categorical variables were performed using Chi-squared test or Fisher’s exact test, while independent two-sample *t*-test was used for comparison of continuous variables. The sensitivity, specificity, positive predictive value (PPV), negative predictive value (NPV), and accuracy of ACR TIRADS, molecular marker testing, and the combined use of both methods were calculated. Statistical analyses were performed with SPSS for Windows (ver. 23.0; IBM, Armonk, NY, USA). A p-value < 0.05 was considered indicative of statistically significant difference.

## Results

The mean age of patients was 47.6 ± 15.5 years (range, 13–74). The mean size of the nodules was 25.8 ± 13.0 mm (range, 4–62). Twenty-nine (67.4%) of the 43 nodules were histologically diagnosed as benign (10 follicular adenomas, 17 hyperplastic nodules, 2 hurthle cell adenomas). Two (4.7%) nodules were borderline thyroid tumors, including one nodule with well-differentiated tumor of uncertain malignant potential (WDT-UMP) and one nodule with follicular tumor of uncertain malignant potential (FT-UMP) (excluded from the statistical analysis because of indeterminate histology). Twelve (27.9%) nodules were malignant including 4 papillary thyroid carcinoma, 7 follicular thyroid carcinoma (FTC) (5 minimally invasive FTC, 2 widely invasive FTC) and 1 medullary thyroid carcinoma. The mean diameter of the benign nodules (25.5 ± 11.9 mm) was not significantly different from that of malignant nodules (26.0 ± 16.6 mm; P = 0.28). The cytological diagnoses for the 43 CITNs were AUS/FLUS (n = 18) and FN/SFN (n = 25). The malignancy rates between AUS/FLUS and FN/SFN were not significantly different [22.2% (4/18) versus 34.8% (8/23), respectively; P = 0.60].

### US features and ACR TIRADS

Malignant nodules had higher rates of solid or almost completely solid composition (91.7% versus 79.3%), hypoechoic (91.7% versus 58.6%), taller-than-wide shape (16.7% versus 3.4%), macro calcification (38.5% versus 29.4%) or punctate echogenic foci (23% versus 20.6%) as compared to benign nodules; however, the between-group differences were not statistically significant (P = 0.62, P = 0.09, P = 0.41, and P = 0.75, respectively). Compared to malignant CITNs, the benign CITNs had higher rates of smooth margin (41.4% versus 8.3%, respectively; P = 0.04), while all nodules with extra-thyroidal extension were malignant. The frequency and risk of malignancy for CITNs based on US features are shown in Table 1.

**Table 1:** Frequency and risk of malignancy for cytological indeterminate thyroid nodules based on ultrasonographic features.

Ultrasonographic feature	Malignant (n = 12)	Benign (n = 29)	Risk of malignancy (%)	P Value
Composition				0.62
Cystic or almost completely cystic	0 (0.0)	0 (0.0)	0	
Spongiform	0 (0.0)	0 (0.0)	0	
Mixed cystic and solid	1 (8.3)	6 (20.7)	14.3	
Solid or almost completely solid	11 (91.7)	23 (79.3)	32.4	
Echogenicity				0.09
Anechoic	0 (0.0)	0 (0.0)	0	
Hyperechoic	0 (0.0)	0 (0.0)	0	
Isoechoic	1 (8.3)	12 (41.4)	7.7	
Hypoechoic	11 (91.7)	17 (58.6)	39.3	
Very hypoechoic	0 (0.0)	0 (0.0)	0	
Shape				0.41
Wider-than-tall	10 (83.3)	28 (96.6)	26.3	
Taller-than-wide	2 (16.7)	1 (3.4)	66.7	
Margin				0.04
Smooth	1 (8.3)	12 (41.4)	7.7	
Ill defined	5 (41.7)	10(34.5)	33.3	
Lobulated or irregular	4 (33.3)	7 (24.1)	36.4	
Extra-thyroidal extension	2 (16.7)	0 (0.0)	100	
Echogenic foci^1^				0.75
None or larger comet tail artifacts	5 (38.5)	16 (50.0)	23.8	
Macro calcification	5 (38.5)	9 (29.4)	35.7	
Peripheral calcification	0 (0.0)	0 (0.0)	0	
Punctate echogenic foci	3 (23.0)	7 (20.6)	30	

^1^ Two subcategories coexisted in 4 nodules. Number in parentheses is percentages.

**Fig 1:**
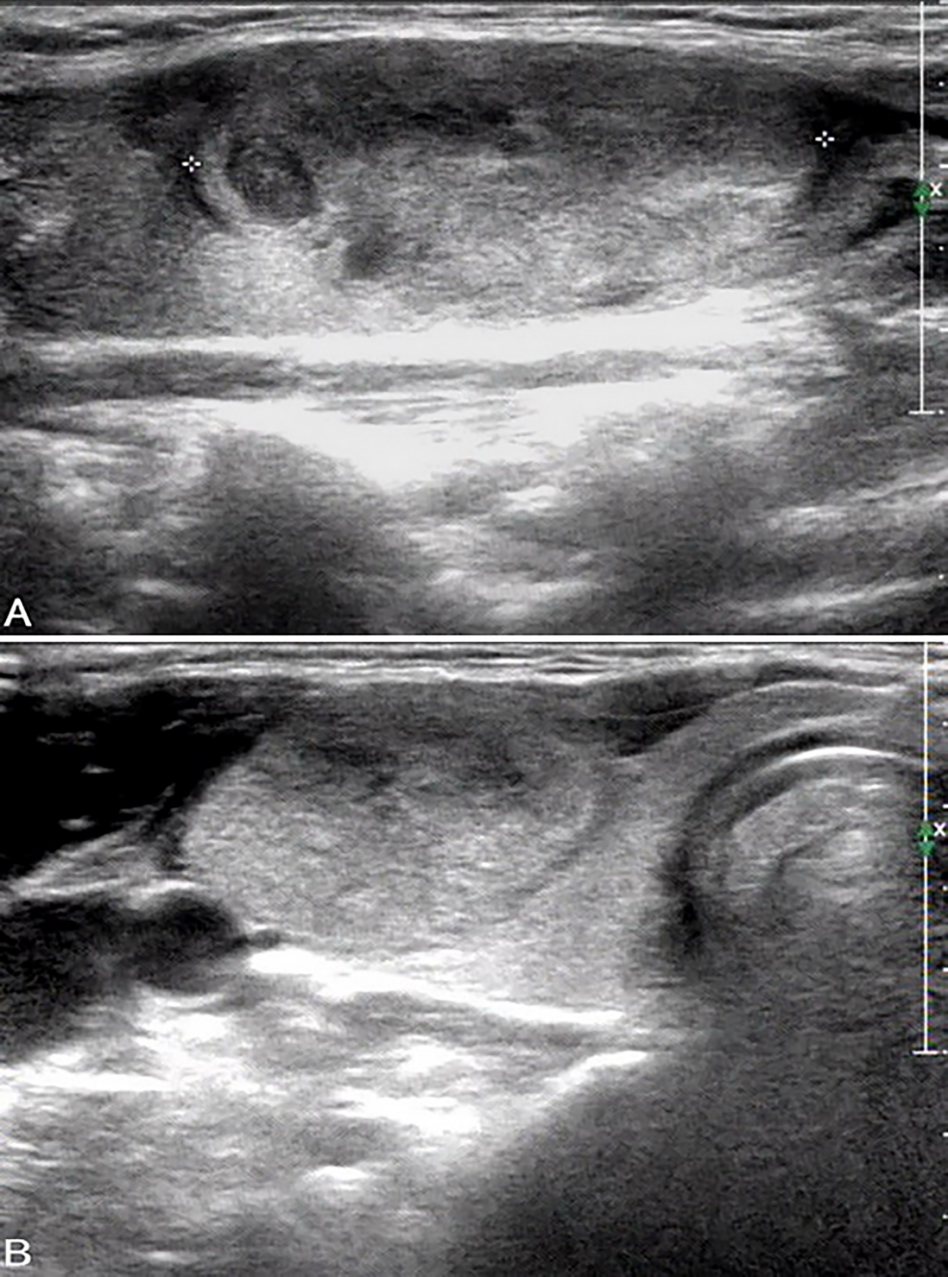
Ultrasound images in a patient with FTC in the right lobe. A, B. Longitudinal (A) and transverse (B) US show an isoechoic solid thyroid nodule (size: 33 mm × 15 mm × 24 mm) with wider-than-tall shape and ill-defined margins. The nodule was classified as ACR TIRADS category TR3. The cytological diagnosis was follicular neoplasm. FNA sample tested negative for *K-RAS* mutation while surgical specimen tested positive.

**Fig 2:**
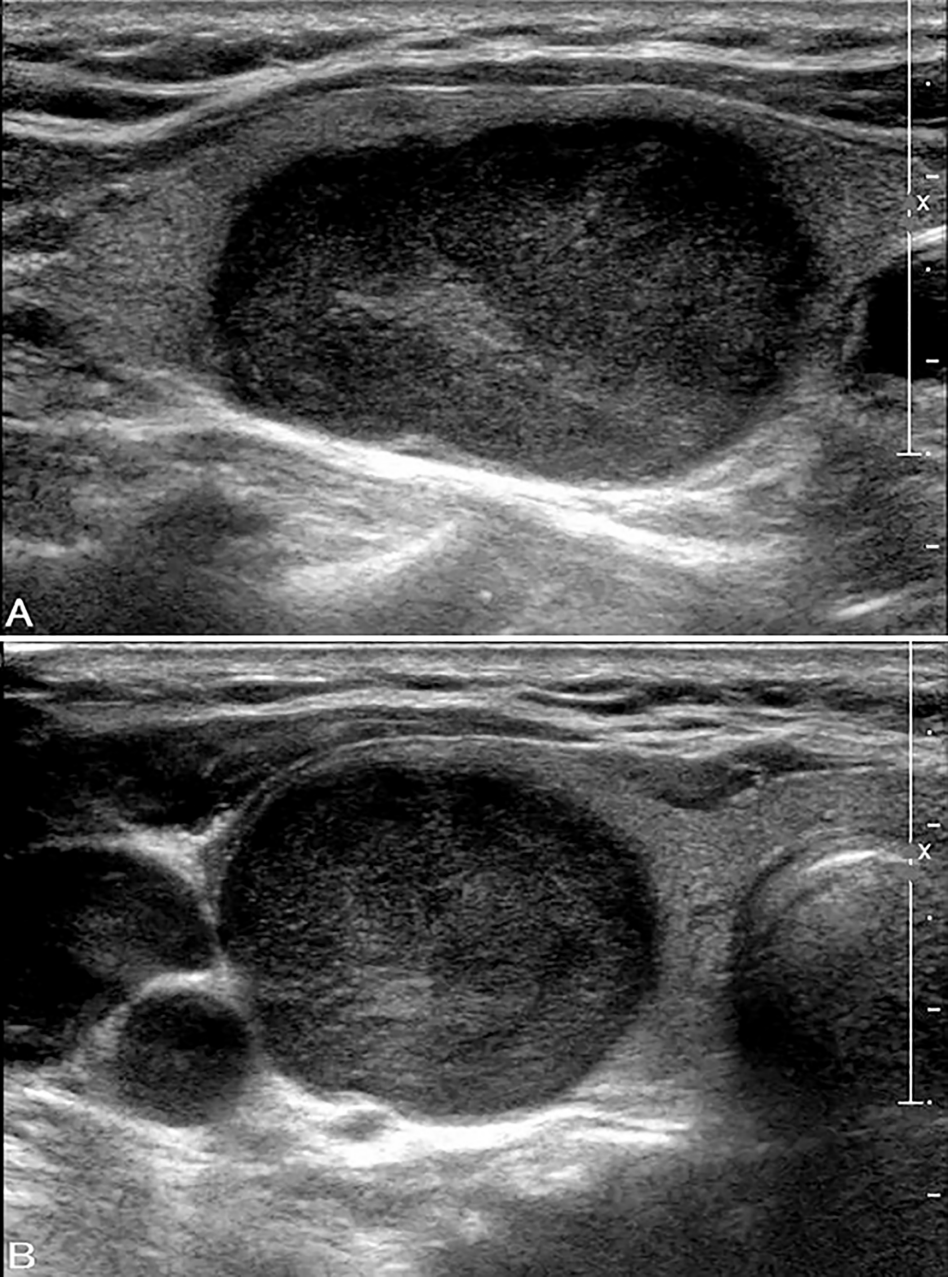
Ultrasound images in a patient with FTC in the right lobe. A, B. Longitudinal (A) and transverse (B) US show a very hypoechoic solid thyroid nodule (size: 32 mm × 19 mm × 24 mm) with wider-than-tall shape and smooth margins. The nodule was classified as ACR TIRADS category TR4. The cytological diagnosis was follicular neoplasm with negative *K-RAS* mutation.

**Fig 3:**
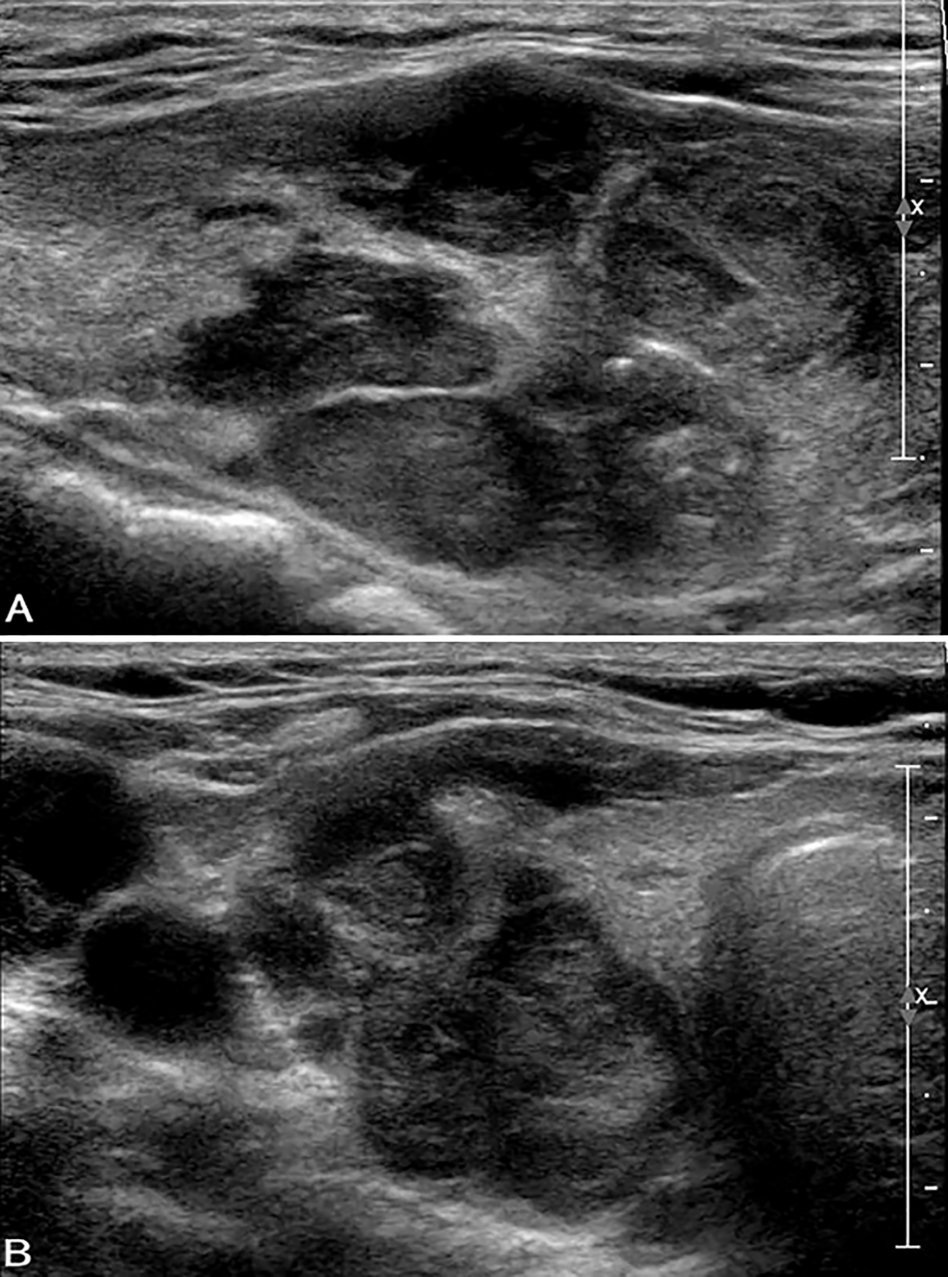
Ultrasound images in a patient with FTC (widely invasive) in the right lobe. A, B. Longitudinal (A) and transverse (B) US show a hypoechoic solid thyroid nodule (size: 43 mm × 24 mm × 29 mm) with wider-than-tall shape and extra-thyroidal extension. Macrocalcification and punctuate echogenic foci are observed. The nodule was classified as ACR TIRADS category TR5. The cytological diagnosis was follicular neoplasm with positive *K-RAS* mutation.

On categorization of CITNs according to ACR TIRADS, the risk of malignancy in ACR TIRADS categories TR2, TR3, TR4, and TR5 was 0%, 40%, 6.7%, and 52.9%, respectively (P = 0.02). TIRADS TR3 and TR5 categories were more prevalent in malignant than benign group (Table 2). The CITNs of category TR3 had a high risk of malignancy (40%), which is high relative to the generally accepted value for ACR TIRADS TR3 nodules. Representative cases are shown in Figs 1–3.

**Table 2:** ACR TIRADS categories of cytological indeterminate thyroid nodules.

ACR TIRADS categories	Points	Malignant (n = 12)	Benign (n = 29)	Risk of malignancy (%)	K-RAS mutation in FNA sample (%)	K-RAS mutation in surgical specimen (%)
TR2	2	0 (0.0)	4 (13.8)	0	0	0
TR3	3	2 (16.7)	3 (10.3)	40	40	60
TR4	4	1 (8.3)	8 (27.6)	6.7	20	33.3
	5	0 (0.0)	2 (6.9)			
	6	0 (0.0)	4 (13.8)			
TR5	7	4 (33.3)	5 (17.2)	52.9	11.8	11.8
	8	2 (16.7)	1 (3.4)			
	9	2 (16.7)	1 (3.4)			
	10	1 (8.3)	1 (3.4)			

Number in parentheses is percentages.

### Tests for molecular markers

All FNA specimens were adequate for successful DNA extraction and detection of molecular markers. Forty-two out of the 43 nodules were negative for *BRAF*^V600E^ mutation, while one *BRAF*-positive nodule was histopathologically diagnosed as PTC.

*K-RAS* mutation was identified in 18.6% (n = 8) of CITNs based on FNA samples and in 25.6% (n = 11) of CITNs based on surgical specimens. The *K-RAS* mutation incidence in FNA sample according to the ACR TIRADS was 0% for TR2, 40% for TR3, 20% for TR4, and 11.8% for TR5 nodules. However, the corresponding incidence based on surgical specimens was 0%, 60%, 33.3%, and 11.8%, respectively (Table 2). CITNs of category TR3 had the highest rate of *RAS*-positivity (40% in FNA sample and 60% in surgical specimens).

We examined the concordance between FNA samples and surgical specimens with respect to *K-RAS* mutation status. Three patients (7.0%) had discordant mutation status; 2 hyperplastic nodules and 1 FTC tested negative for *K-RAS* mutation in FNA samples but tested positive in surgical specimens. For surgical specimens, after excluding one case of WDT-UMP with *RAS*-positive mutation, *K-RAS* mutations were detected in 4 out of the 12 (33.3%) malignant nodules (1 PTC and 3 FTCs) and in 6 out of 29 (20.7%) benign nodules, including 1 follicular adenoma, 1 hurthle cell adenoma, and 4 hyperplastic nodules. Coincidentally, *K-RAS* mutated malignancy was more frequently found in FN/SFN than in AUS/FLUS (50% vs. 0%, respectively; P < 0.01). The results of *K-RAS* mutation according to Bethesda category are presented in Table 3.

**Table 3:** Molecular analysis based on Bethesda category.

Molecular analysis	Bethesda III (AUS/FLUS) (n = 18)		Bethesda IV (FN/SFN) (n = 23)	
	Benign (n = 14)	Malignant (n = 4)	Benign (n = 15)	Malignant (n = 8)
BRAF^V600E^ mutation	0 (0.0)	1 (25.0)	0 (0.0)	0 (0.0)
K-RAS mutation in FNA sample	2 (14.3)	0 (0.0)	2 (13.3)	3 (37.5)
K-RAS mutation in surgical specimen	2 (14.3)	0 (0.0)	4 (26.7)	4 (50)

Number in parentheses is percentages

For *K-RAS* positive malignant CITNs based on surgical specimen, 2/4 (50%) exhibited extra-thyroidal extension (ETE), 1/4 (25%) had pathologically confirmed central and lateral lymph node metastasis, while none showed distant metastasis. For *K-RAS* negative malignant CITNs, 3/8 (37.5%) had central lymph node metastasis, 1/8 (12.5%) had lateral lymph node metastasis, while none had ETE or distant metastasis. There was no significant difference in aggressiveness between the two groups (ETE, P = 0.17; lymph node metastasis, P = 0.84).

### Diagnostic performance

The diagnostic performance including sensitivity, specificity, PPV, NPV, and accuracy according to ACR TIRADS, *K-RAS* mutation analysis, and the combination are shown in Table 4. The specificity of *K-RAS* mutation to detect malignancy in CITNs was 86.2%, with a low sensitivity of 25%. When considering categories TR2 and TR3 as negative test outcomes and categories TR4 and TR5 as positive test outcomes, the diagnostic performance of ACR TIRADS were as follows: sensitivity 83.3%, specificity 24.1%, PPV 31.3%, NPV 77.8%, and accuracy 41.5%. On using a higher threshold for positivity (ACR TIRADS category TR5), the sensitivity, specificity, PPV, NPV, and accuracy for malignancy were 75.0%, 72.4%, 52.9%, 87.5%, and 73.2%, respectively. When ACR TIRADS was combined with *K-RAS* mutation analysis, combination of *K-RAS* and ACR TIRADS (category TR5 as threshold) compared to *K-RAS* mutation improved the sensitivity (83.3% vs. 25.0%, P < 0.05) and the NPV (89.5% vs. 73.5%, P < 0.05) for diagnosis of CITNs. However, *K-RAS* and ACR TIRADS (category TR5 as threshold) combined did not yield a notable improvement in sensitivity (83.3% vs. 75.0%, P > 0.05) and NPV (89.5% vs. 87.5%, P > 0.05) compared with ACR TIRADS alone.

**Table 4:** Diagnostic performance of K-RAS mutation, ACR TIRADS and combination.

Diagnostic Modality	Sensitivity (95% CI)	Specificity (95% CI)	PPV (95%CI)	NPV (95%CI)	AC (95%CI)
K-RAS mutation in FNA sample	25.0 (5.5–57.2)	86.2 (68.3–96.1)	42.9 (16.5–74.1)	73.5 (66.0–79.9)	68.3 (51.9–81.9)
TIRADS 1^1^	83.3 (51.6–97.9)	24.1 (10.3–43.5)	31.3 (24.7–38.6)	77.8 (45.8–93.5)	41.5 (26.3–57.9)
TIRADS 2^2^	75.0 (42.8–94.5)	72.4 (52.8–87.3)	52.9 (36.4–68.8)	87.5 (71.9–95.0)	73.2 (57.1–85.8)
Combination 1	91.7 (61.5–99.8)	20.7 (8.0–39.7)	32.4 (27.1–38.1)	85.7 (44.6–97.8)	41.5 (26.3–57.9)
Combination 2	83.3 (51.6–97.9)	58.6 (38.9–76.5)	45.5 (33.5–57.9)	89.5 (69.8–96.9)	65.9 (49.4–79.9)

^1^ TR4 was taken as the diagnostic threshold.

^2^ TR5 was taken as the diagnostic threshold.

Combination 1 = K-RAS mutation +TIRADS 1; Combination 2 = K-RAS mutation +TIRADS 2. PPV (positive predictive value); NPV (negative predictive value); AC (accuracy)

## Discussion

Indeterminate thyroid cytology is a gray zone wherein the cytological evaluation is inconclusive. Up to 25% of thyroid FNAs cannot be categorized as either benign or malignant according to the Bethesda System for Reporting Thyroid Cytopathology [5]. The risk of malignancy is varied, ranging from 5%–15% for AUS/FLUS to 15%–30% for FN/SFN diagnosed by examination of surgical specimens [5]. Therefore, tools that help identify or exclude malignant CITNs may facilitate appropriate management and treatment. In recent years, molecular marker testing has become available as an aid to refine the diagnosis of CITNs. Even though molecular markers help in risk stratification of nodules with indeterminate cytology [10–11], there is no current consensus on the role of *RAS* mutation as a predictor of malignancy. In the present study, we evaluated the US features according to ACR TIRADS and examined the frequency of *K-RAS* mutation in CITNs. The objective was to determine whether the use of ACR TIRADS in combination with *K-RAS* mutation status can help provide an optimal management decision for thyroid nodules with indeterminate cytology.

In a multicenter prospective study by Ha et al., presence of microcalcification, taller-than-wide tumors, and spiculated/microlobulated margins were independent predictors of malignancy [12]. However, these suspicious US features were not significantly associated with follicular thyroid carcinoma. In this study, a greater percentage of malignant nodules were wider-than-tall, had no punctuate echogenic foci, or presented with larger comet-tail artifact. Theoretically, thyroid nodules can be stratified using ACR TIRADS according to malignancy risk based on suspicious US features [4]. In the present study, use of ACR TIRADS for risk stratification of CITNs using TR5 as the diagnostic threshold was associated with 75.0% sensitivity and 72.4% specificity. Suspicious US features did not show good performance in predicting malignancy in CITNs.

Generally, the risk of malignancy tends to increase with advancing ACR TIRADS category. However in the present study, the malignancy risk of category TR3 lesions was higher than the indicated malignancy risk of 4.8% [95% confidence interval (CI), 3.4–6.5%] for TR3 proposed by ACR TIRADS [13], as well as the malignancy risk of category TR4 thyroid nodules. Some cases of FTCs among malignant nodules may be the underlying cause of increased malignancy risk. Two malignant CITNs categorized as TR3 were FTCs with no suspicious US features, which is an indication for active surveillance or UG-FNA follow-up; however, in the present study, these patients were referred for surgery owing to pressure symptoms or increase in size. We believe that the risk of malignancy in CITNs with category TR3 based on surgical specimens may be higher than that of ACR TIRADS TR3 nodules reported in previous reports. We believe that US follow-up is insufficient for CITNs with ACR TIRADS category TR3; however, our findings should be validated in a larger study.

Ancillary molecular analyses for common somatic mutations of thyroid cancer have been developed to improve the diagnostic accuracy of FNA for CITNs [10–11, 14–18]. Follicular thyroid tumors commonly harbor *RAS* mutations, and in general, the presence of *RAS* mutation is associated with good prognosis. In the present study, *K*-*RAS* mutations were identified in 18.6% of the CITNs; this percentage is comparable to that reported from a large cohort study by Nikiforov et al. [9], but is lower than that reported from other series [19–21]. Positive *K-RAS* mutation may facilitate clinical management of indeterminate thyroid cytology, which is associated with a moderate risk of malignancy according to previous reports [5]; this represents a dilemma in treatment decision-making. Ten *K-RAS* mutated surgical specimens were found in our study; of these, 2 were diagnosed as AUS/FLUS and 8 as FN/SFN. In addition, 50% (4/8) of *K-RAS* mutated FN/SFN were found to be malignant at surgery. Therefore, *K*-*RAS* mutation was more frequently associated with malignancy in FN/SFN as compared to AUS/FLUS. This indicates that resection may be a better approach for such patients.

As suggested by a previous study [22], *RAS* mutation-positive tumors have limited aggressive behavior, including extra-thyroidal extension, lymph node metastasis, or distant metastasis. Our study also demonstrates that *RAS*-positive thyroid carcinoma is no more aggressive than the wild-type malignancy. However, ETE is more frequently detected in *RAS*-positive CITNs. The low detection rate of *BRAF*^V600E^ mutation in this study is similar to that in a previous study [23] in which 5.6% (1/18) of patients with AUS/FLUS and 0% (0/25) of those with FN/SFN had *BRAF*^V600E^ mutation. Although with negative *BRAF* mutations, we could not exclude that poor tumor behavior is associated with the coexistence of TERT promoter mutation, which was not tested in this study.

Our study affirms the results of previous studies that *RAS* mutation has a low sensitivity and a high specificity for detection of malignant CITNs [11, 19–20]. In this study, *K*-*RAS* mutation was found in 21.1% of benign CITNs, and a number of malignant CITNs did not harbor *K-RAS* mutation, which may be associated with the low sensitivity. Because of low sensitivity, *K-RAS* mutation is often recommended as a supplemental aid to management of CITNs. We found that use of ACR TIRADS criteria in conjunction with *K-RAS* mutation increased the sensitivity of *K-RAS* mutation from 25% to 83.3%. In other words, use of ACR TIRADS in combination with *K-RAS* mutation increased the sensitivity by 58.3% as compared to that of *K-RAS* mutation alone. We observed that the NPV of *K-RAS* mutation alone for malignant diagnosis was 73.5%, which increased to 89.5% when used in combination with ACR TIRADS; this suggests that combined use of both methods may help avoid unnecessary thyroidectomy. The results of our small-scale preliminary study indicate that use of *K-RAS* analysis in conjunction with ACR TIRADS may be potentially useful for excluding CITNs that require resection. Therefore, follow-up may be recommended for patients with category TR2, TR3, and TR4 CITNs who test negative for *K*-*RAS* mutation. However, for CITNs with ACR TIRADS scores >6, further work up should be performed owing to the high risk of malignancy.

In this study, discordant false negative results for *K*-*RAS* status between FNA samples and surgical specimens were identified in samples from 3 (7.0%) patients. The false negative results may be due to insufficient number of tumor cells in the leftover material after preparation of cytological smears [24]. However, none of the cases in our study were affected by insufficient and/or poor quality DNA. Studies have shown that some genetic mutations are inhomogeneously distributed in tumor cells and that the mutated allele percentage is liable to vary within the tumor [25–26]. This may be attributable to greater heterogeneity among larger nodules with respect to mutation when compared with nodules of small size. Since only a portion of the tumor is sampled during FNA, the FNA results are less likely to sufficiently represent the surgical results. The issue of tumor heterogeneity or focal presentation suggests that due caution should be exercised during clinical decision-making solely based on molecular markers.

Some limitations of our study should be acknowledged. First, our sample size was relatively small. In contrast to *BRAF* mutation, *K*-*RAS* mutation is only available for CITNs. As a cancer referal center, indeterminate cytological diagnosis is observed in up to 17% of thyroid FNAs performed at our institution. Most patients with CITNs are suggested to undergo active surveillance or repeat FNA. Second, for precise identification, we only included nodules for whom surgical results were available. So the relatively higher malignancy rate among CITNs of category TR3 may not be representative. In addition, small sample size tends to lead to overestimation of malignancy rate. Third, owing to the retrospective study design, our results may have been affected by selection bias. Additionally, in the present study, only *K-RAS* mutaion testing was performed. The cost-effective difference between limited mutation analysis compared to full gene expression classifer or next generation sequencing is needs to be further investigated. Although our initial results are promising, further large-scale prosepctive studies are required to validate the role of *K*-*RAS* mutation in routine clinical use.

## Conclusion

In conclusion, CITNs with ACR TIRADS category TR3 were unexpectedly associated with a high risk of malignancy. Patients with *K-RAS* mutation-positive FN/SFN nodules exhibited a 50% risk of malignancy; therefore, resection should be considered in such cases to obtain a definitive diagnosis. In this study, use of ACR TIRADS in combination with *K*-*RAS* mutation was associated with an 89.5% NPV for malignant CITDs. Our results suggest that use of a combination of TIRADS and *K-RAS* mutation status can improve thyroid management and help avoid unnecessary surgery.
